# A QRS Detection and R Point Recognition Method for Wearable Single-Lead ECG Devices

**DOI:** 10.3390/s17091969

**Published:** 2017-08-26

**Authors:** Chieh-Li Chen, Chun-Te Chuang

**Affiliations:** 1 Department of Aeronautics and Astronautics, National Cheng-Kung University, Tainan 70101, Taiwan; chiehli@mail.ncku.edu.tw; 2 Industrial Technology Research Institute, Tainan 70101, Taiwan

**Keywords:** ECG, QRS detection, heartbeat detection, mobile healthcare, IoT, wearable device, edge computing

## Abstract

In the new-generation wearable Electrocardiogram (ECG) system, signal processing with low power consumption is required to transmit data when detecting dangerous rhythms and to record signals when detecting abnormal rhythms. The QRS complex is a combination of three of the graphic deflection seen on a typical ECG. This study proposes a real-time QRS detection and R point recognition method with low computational complexity while maintaining a high accuracy. The enhancement of QRS segments and restraining of P and T waves are carried out by the proposed ECG signal transformation, which also leads to the elimination of baseline wandering. In this study, the QRS fiducial point is determined based on the detected crests and troughs of the transformed signal. Subsequently, the R point can be recognized based on four QRS waveform templates and preliminary heart rhythm classification can be also achieved at the same time. The performance of the proposed approach is demonstrated using the benchmark of the MIT-BIH Arrhythmia Database, where the QRS detected sensitivity (Se) and positive prediction (+P) are 99.82% and 99.81%, respectively. The result reveals the approach’s advantage of low computational complexity, as well as the feasibility of the real-time application on a mobile phone and an embedded system.

## 1. Introduction

**Figure 1:**
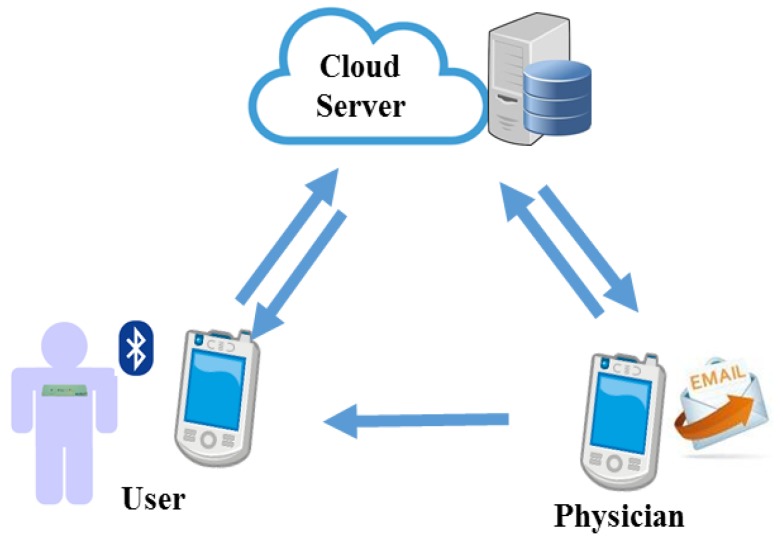
Mobile healthcare system.

A mobile healthcare system, as shown in Figure 1, has been one popular application of the internet of things (IoT) in recent years. Different from the traditional measurement devices, wearable devices can monitor long-term physiological signals and upload data to the cloud via a wireless communication system [1,2,3]. Therefore, low computation complexity and high efficiency algorithms become important issues in mobile healthcare systems.

**Figure 2:**
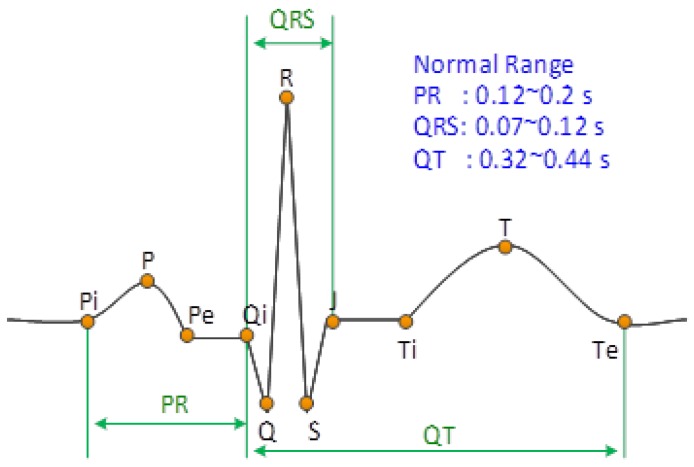
Typical ECG waveform of a cardia cycle (normal sinus rhythm in Lead II).

For a remote electrocardiogram (ECG) monitoring application, QRS detection is a preliminary step for detecting the heartbeat for the subsequent rhythm classification, so a high QRS detection rate method is the most significant part of the ECG analysis algorithm. Figure 2 shows the feature points (P, Q, R, S, and T) of a typical ECG waveform of a cardiac cycle in lead II. However, the measured ECG waveforms are usually different from one another due to motion artifact effects or abnormal rhythms [4], which leads to difficulty in detecting QRS. In the literature, there were numerous QRS detection methods proposed based on quadratic energy [5,6,7,8], the Shannon energy envelope [9,10], wavelet transform [11,12,13], adaptive threshold [14,15], and adaptive filter [16]. Based on a two-stage median filter and independent component analysis (ICA), approaches [17,18,19] were utilized in the signal pre-processing stage to eliminate the baseline wandering and waveform distortion caused by motion artifacts. Besides, recognizing Q, R, and S feature points for both typical and atypical QRS waveforms is also an important issue for clinical implications. There are nine pre-specified categories of QRS waveforms [20] applied to recognize reasonable R points. In addition, an adaptive mathematical morphology approach was proposed to extract the real R points [21]. Generally, most of the foregoing methods exhibited a good performance in QRS detection; however, issues relating to power efficiency and implementation simplicity are the main concerns for embedded system and mobile applications.

This study proposes a real-time QRS detection and R point recognition method with a high accuracy but very low computational complexity. It is achieved by the enhancement of QRS segments with the restraining of P and T waves. The QRS recognition is carried out based on four typical QRS waveform templates. The performance of the proposed method is verified using the MIT-BIH Arrhythmia Database and was implemented in a mobile phone and an embedded system (ARM Cortex M3), respectively, to demonstrate the real-time performance.

The paper is organized as follows. Section 2 presents the proposed methodology. The detected result according to the MIT-BIH Arrhythmia Database using the proposed method is demonstrated in Section 3. Section 4 presents the implementation of the proposed method on a mobile phone and an embedded system, as well as the comparison with a heart rate sensor product. Section 5 is the conclusion.

## 2. Methodology

**Figure 3:**
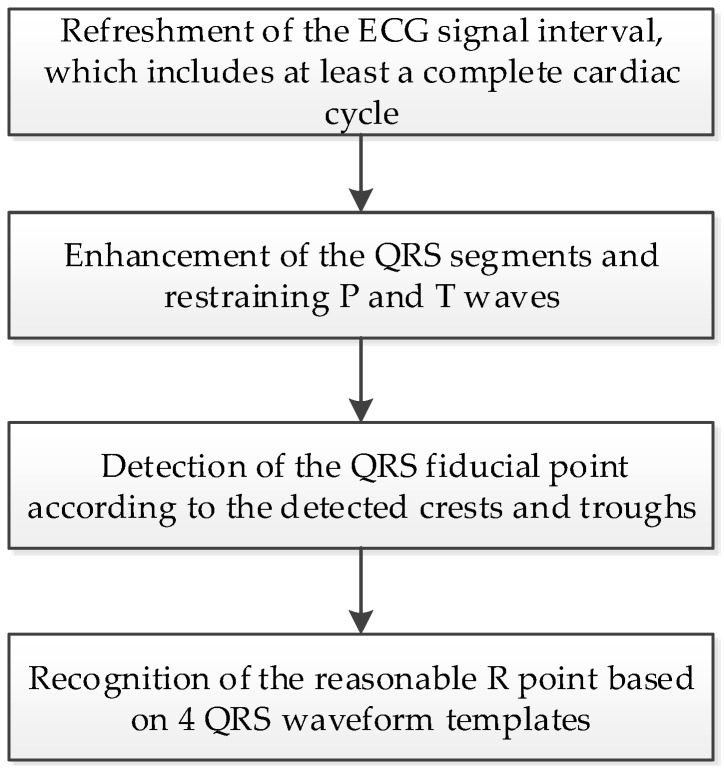
The procedures of the proposed real-time QRS detection and R point recognition method.

The procedure of the proposed QRS detection and R point recognition method can be divided into four steps, as shown in Figure 3. The detailed process for each step is as follows.

### 2.1. Refreshment of the ECG Signal

**Figure 4:**
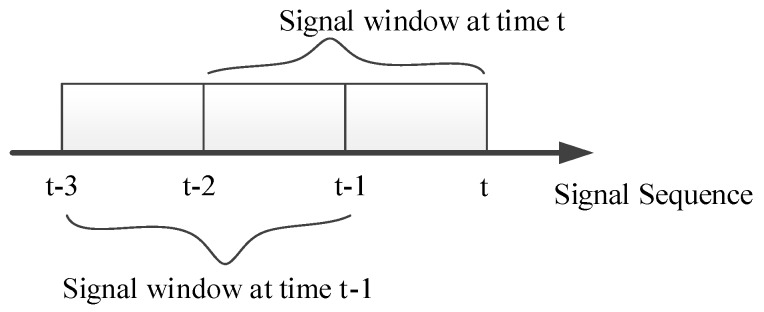
ECG signal refreshment.

In most ECG devices, the monitoring heart rate range is from 30 to 240 beats per minute (bpm); therefore, there is at least one complete cardiac cycle within a two-second time interval. For this reason, a sliding window of two seconds is used to capture the ECG signal and the obtained ECG signal is refreshed every second, as shown in Figure 4.

### 2.2. Signal Enhancement

**Figure 5:**
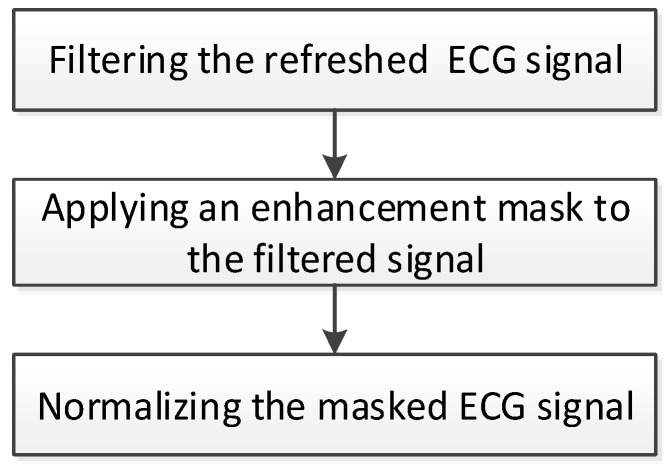
Signal transform procedure.

In a normal sinus rhythm (NSR), the QRS wave usually has a sharp shape in lead II, as shown in Figure 2. However, for a wearable device such as a 24-h ECG recorder (holter), motion artifacts and abnormal rhythms bring about baseline wandering, amplitude variation, and atypical waveforms. This study proposed a signal transformation procedure, as shown in Figure 5, to eliminate motion artifact effects, and enhance the QRS segments, as well as restrain P and T waves. The refreshed ECG signal is filtered using a band-pass filter with a bandwidth between 0.5 and 17 Hz to eliminate noise and motion artifacts.

Then, the enhancement of the QRS segment whilst restraining P and T waves is achieved by applying the following mask:

(1) $$S(n)=\sum_{j=-k}^{k}M(k+j)E(n+j)$$

where *M* represents the enhancement mask and:

(2) $$\{\begin{array}{c} M(n)=-1,\;n=0∼k-1\;\\ M(n)=2*k,\;n=k\\ M(n)=-1,\;n=k+1∼2k \end{array}$$

*E* represents the filtered ECG signal and *S* represents the masked ECG signal. The zero sum of M’s entries will eliminate baseline wandering. Subsequently, the QRS amplitude variation is eliminated by normalizing the masked ECG signal to 1.

**Figure 6:**
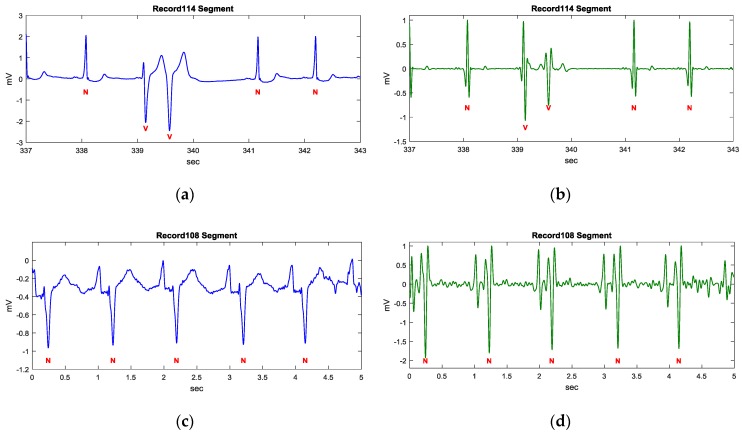

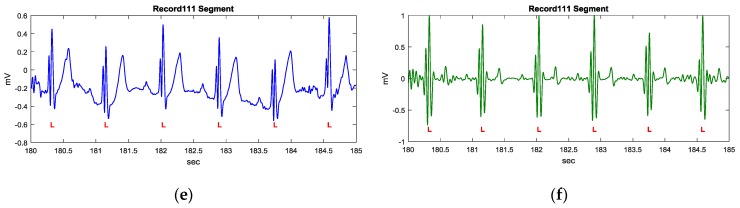
Signal enhancement using the proposed transformation. (**a**) Filtered ECG signal of the Record 114 segment; (**b**) Transformed signal of the Record 114 segment by the proposed transformation; (**c**) Filtered ECG signal of the Record 108 segment; (**d**) Transformed signal of the Record 108 segment by the proposed transformation; (**e**) Filtered ECG signal of the Record 111 segment; (**f**) Transformed signal of the Record 111 segment by the proposed transformation.

Figure 6 demonstrates three examples of different ECG rhythms provided by the MIT-BIH arrhythmia database using *k* = 1, where the annotation N represents a normal sinus rhythm (NSR), V represents a premature ventricular contract rhythm (PVC), and L represents a left bundle branch block rhythm (LBBB). Figure 6a,c,e illustrate the filtered ECG signal of Record 114, 108, and 111 segments, respectively, using a low pass filter with a 40 Hz cutoff frequency. The Record 114 segment is of NSR and PVC rhythms. The transform result shown in Figure 6b illustrates a restraining of P and T waves and an enhancement of QRS complexes. The Record 108 segment is of an atypical NSR rhythm, which contains bigger P waves and inverse QRS complexes. The transformed result demonstrates that the T waves are restrained, while bigger P waves and QRS segments are enhanced, as shown in Figure 6d. The Record 111 segment is of an LBBB rhythm, which contains fork-like QRS complexes and relatively tall T waves. Figure 6e shows that the fork-like QRS complexes are enhanced while retaining P and T waves.

In the above examples, most of P and T waves are restrained and QRS segments are enhanced by the proposed signal transformation. Besides, the amplitudes in the transformed signal are also normalized to 1 to eliminate the amplitude difference from person to person. As a result, the crests and troughs can be detected using static thresholds.

### 2.3. The QRS Fiducial Point Detection

For a typical lead II ECG waveform as shown in Figure 2, the feature intervals, such as the PR interval, QRS duration, QRS amplitude, and QT interval, have normal ranges. Therefore, the searching range is defined as 0.3 s and the minimum amplitude for detecting the QRS complex is defined as 0.5 mV in this study.

**Figure 7:**
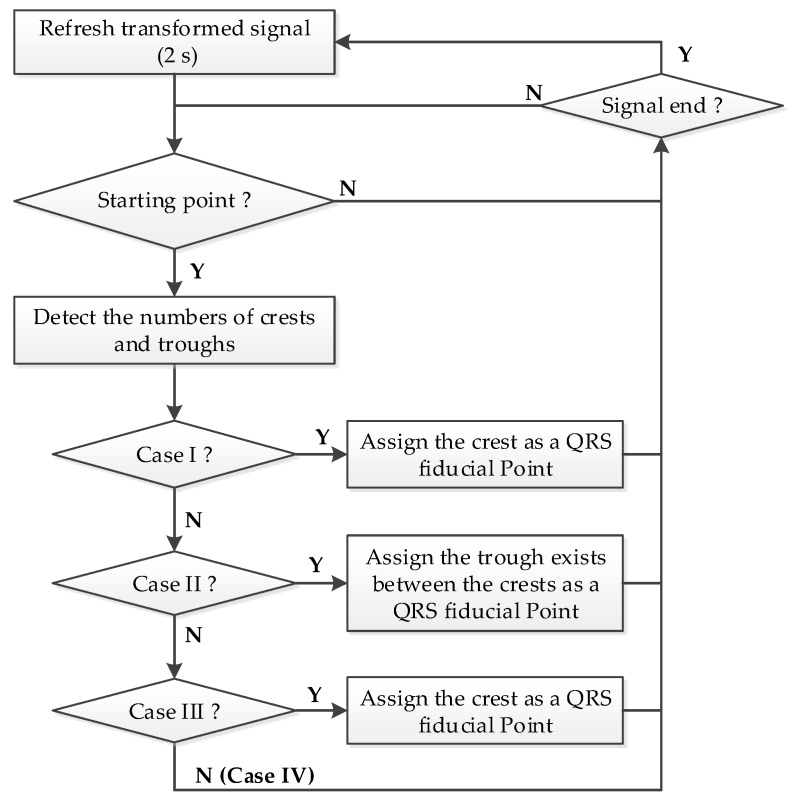
The proposed procedure of detecting the QRS fiducial point.

If the magnitude range of the refreshed ECG signal segment is higher than 0.5 mV, the procedure shown in Figure 7 will activate to detect the QRS fiducial point. Due to the transformed signal being normalized to 1, a positive threshold of 0.22 is used to detect the crests, and a negative threshold of −0.2 is used to detect the troughs, respectively. The first instance of a transformed ECG signal of a value higher than the positive threshold is marked as the starting point for QRS detection.

- Case I:
  If only one crest and only one trough exist within the specified searching range (0.3 s), the instance corresponding to the crest is defined as the QRS fiducial point and search for the next starting point after the detected trough.
- Case II:
  If two crests are within the searching range and one trough exists between the crests, then the trough is defined as the QRS fiducial point and search for the next starting point from 0.12 s after the detected trough is for. It is noted that a normal QRS waveform has a duration of 0.12 s.
- Case III:
  If there is only one crest with a value higher than another threshold of 0.52 within the searching range, then the crest is defined as the QRS fiducial point and search for the next starting point from 0.12 s after the detected crest. This case is a special case which can be referred to the MIT-BIH arrhythmia database Record 104 and 107.
- Case IV (Case otherwise):
  If it is not case I, case II, or case III, then search for the next starting point corresponding to the next crest.

**Figure 8:**
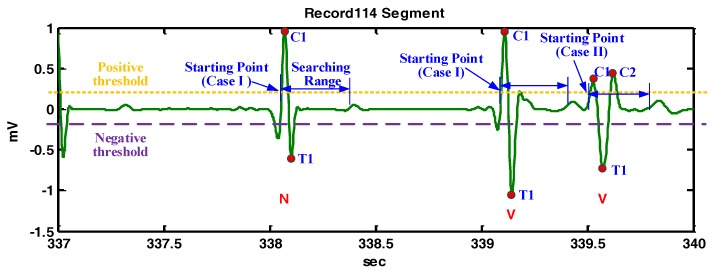
The demonstration of QRS fiducial point detection for the Record 114 segment.

Figure 8 demonstrates the detailed detection procedures of the example shown in Figure 6b. For the starting point at 338.1 s, there is only one crest and only one trough within the searching range, so the crest is defined as a QRS fiducial point. For the next starting point at 339.5 s, there are two crests within the searching range and one trough exists between the crests, so the trough is defined as the QRS fiducial point.

**Figure 9:**
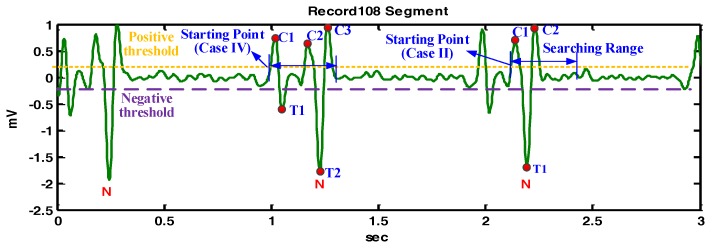
The demonstration of QRS fiducial point detection for the Record 108 segment.

Figure 9 demonstrates the detailed detection procedures of the example shown in Figure 6d. For the starting point at 1 s, there are three crests and two troughs within the searching range, so that the starting point is redefined as the starting point of the next crest. The detection corresponding to the starting point of the next crest is similar to that of the starting point at 2.1 s. In this example, QRS fiducial points are determined effectively in the QRS segment, even under the influence of big P waves.

**Figure 10:**
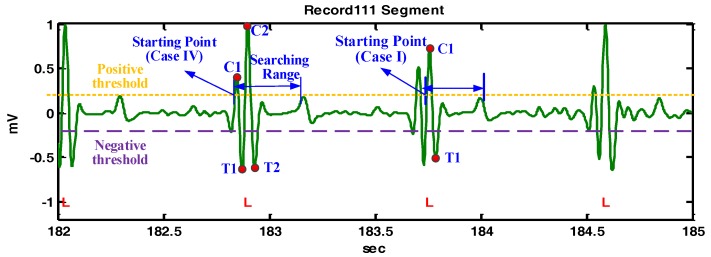
The demonstration of QRS fiducial point detection for the Record 111 segment.

Figure 10 demonstrates the detailed detection procedures of the example shown in Figure 6f. For the starting point at the first detected crest at 182.9 s, there is no second crest and no second trough, so the starting point is redefined as the starting point of the next crest. Subsequently, case I will be applied and C2 becomes the QRS fiducial point. A similar case is also illustrated at 183.7 s. It should be noted that the detected QRS fiducial point in the transformed signal may not correspond to the real R point in the ECG signal and an R point recognition procedure is described as the following.

### 2.4. Recognition of the R Point

**Figure 11:**
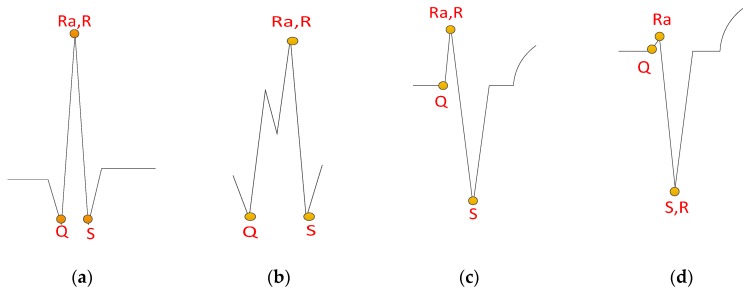
QRS waveform templates of four desired recognition features: (**a**) normal QRS waveform; (**b**) fork-like crests; (**c**) deep S wave with small R wave; (**d**) deep S wave with tiny or hidden R wave.

To recognize a reasonable R point according to the detected QRS fiducial point, four QRS waveform templates are proposed, as shown in Figure 11. The strategy of finding those features includes first searching for the S point from the fiducial QRS point, then searching for the Ra, Q, and R points successively, where Ra is the approximate R point, in order to find a reasonable R point. The details of each step are as follows.

#### 2.4.1. S Point Detection

In general, a normal QRS segment range is less than 0.12 s. To identify the corresponding Q, R, and S points for a given QRS fiducial point, it is reasonable to use a searching time interval span of 0.24 s centering at the fiducial point. The S point detection procedure is described as follows:

- Step 1:
  For a given QRS fiducial point, determine the maximum and average amplitude within the 0.24 s searching span of the ECG signal.
- Step 2:
  Search a crest of amplitude higher than one half of the maximum amplitude after the QRS fiducial point.
- Step 3:
  If there is a detected crest, the S start searching point is assigned as the detected crest. Otherwise, the S start searching point is assigned as the QRS fiducial point.
- Step 4:
  The S point is assigned as the point of amplitude less than the average amplitude with a local minimum slope after the S start searching point.

#### 2.4.2. Ra Point Detection

With a detected S point and its corresponding searching time span, the Ra point is determined according to the following. If there is at least one crest ahead of the detected S point with its amplitude higher than one half of the maximum within the searching range, the Ra point is assigned as the maximum amplitude point. Otherwise, the Ra point is assigned at the same point of S.

#### 2.4.3. Q Point Detection

With a detected Ra point and the corresponding searching time span, the Q point is identified by the following procedure.

- Step 1:
  Search a crest of amplitude higher than one half of the maximum amplitude ahead of the detected Ra point.
- Step 2:
  If there is a detected crest, the Q start searching point is assigned as the detected crest. Otherwise, the Q start searching point is assigned as the Ra point.
- Step 3:
  The Q point is assigned as the point of amplitude less than the average amplitude with a local minimum slope ahead of the Q start searching point.

#### 2.4.4. R Point Detection

With detected Ra and S points, the R point is determined by the following rules:

- Case 1:
  If the Ra point and the S point are at the same position, then assign the R point at point S.
- Case 2:
  If the amplitude between the Ra point and the Q point is less than a quarter of the amplitude between Ra and S, then assign the R point at point S.
- Case 3:
  Otherwise, assign the R point at the Ra point.

## 3. Result and Discussion

### 3.1. QRS Detection and R Point Recognition Result

**Figure 12:**
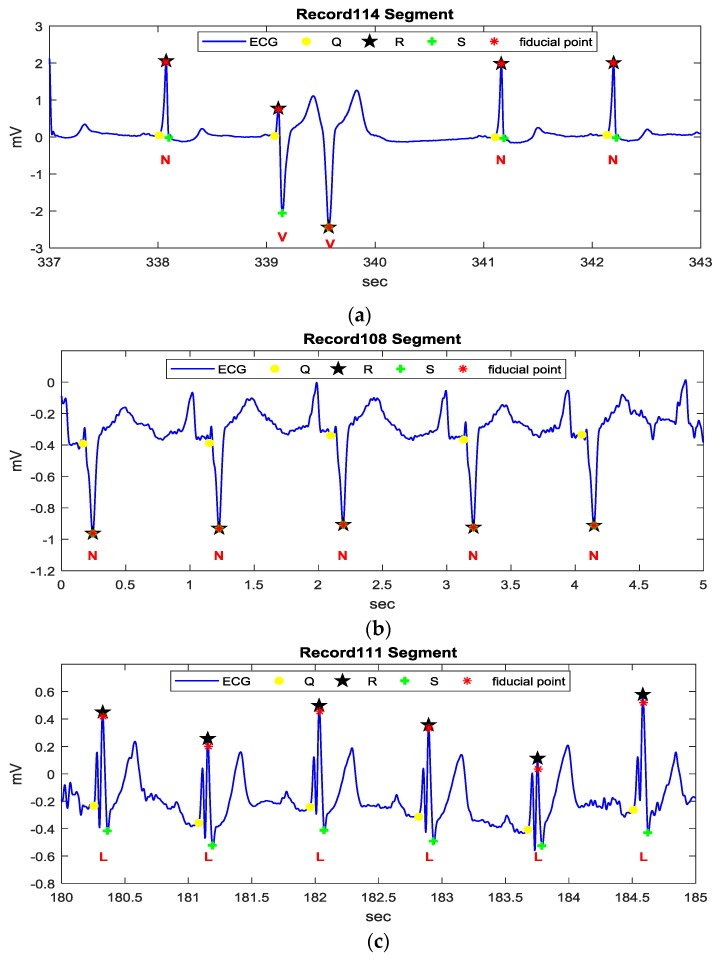
The detected QRS fiducial points and R points using the proposed method: (**a**) recognized feature points for the Record 114 segment; (**b**) recognized feature points for the Record 108 segment; (**c**) recognized feature points for the Record 111 segment.

Figure 12 illustrates the detected QRS fiducial points and the recognized feature points (Q, R, and S) of the three examples, based on the four proposed QRS waveform templates, shown in Figure 11. Figure 12a illustrates the correctly detected features defined in Figure 11a,c,d. Figure 12b illustrates the correctly detected features defined in Figure 11d. Figure 12c illustrates the correctly detected features defined in Figure 11c. The results in Figure 12 illustrate that different rhythms can be successfully recognized by the proposed method.

**Figure 13:**
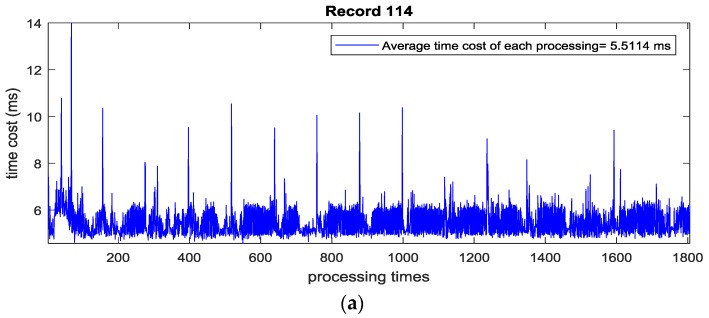

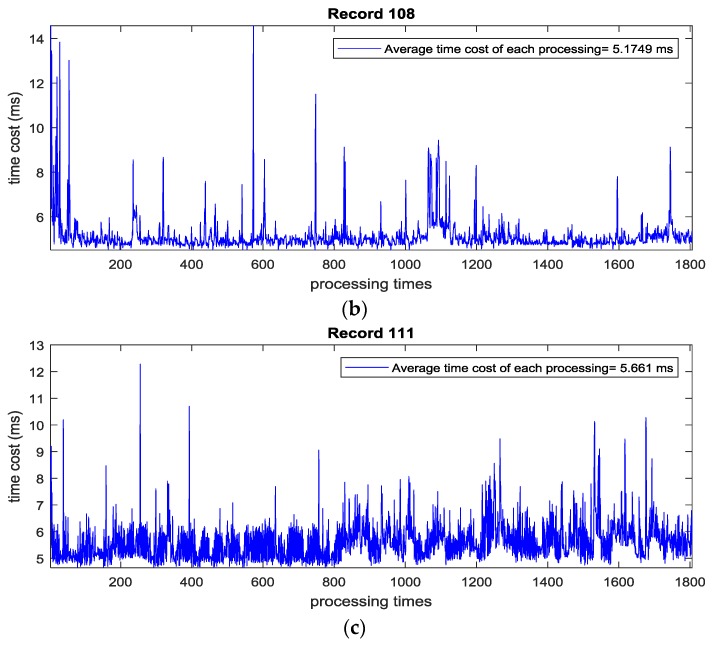
The time cost for processing each two-second length ECG signal: (**a**) Time cost of each processing in Record 114; (**b**) Time cost of each processing in Record 108; (**c**) Time cost of each processing in Record 111.

Before implementing the algorithm in an embedded system, the time cost of each processing for the two-second length ECG signal from the MIT-BIH arrhythmia database (360 Hz) is evaluated using Matlab in a Win 7 PC with Intel i5 dual core CPU. Through test runs of the above examples, the maximum time cost of processing once is less than 15 milliseconds and the average time cost of each processing is less than 6 milliseconds, which implies that the algorithm is portable for a real-time embedded system in Figure 13.

### 3.2. Benchmarking Study Using MIT-BIH Arrhythmia Database

The MIT-BIH arrhythmia database contains 48 half-hour excerpts of two-channel ambulatory ECG recordings. The recordings were digitized at 360 samples per second per channel with an 11-bit resolution over a 10 mV range [22]. The standard of ANSI/AAMI/ISO EC57:1998/(R)2008 claims that the QRS detection algorithm has to supply the reporting of statistics from the MIT-BIH arrhythmia database [23].

To evaluate the performance of the proposed method, the commonly used detector performance measures are applied and defined as follows.

Sensitivity (Se) indicates the fraction of events which are detected:

(3) $$\mathrm{Se}=\frac{TP}{TP+FN}\times 100\%$$

Positive prediction (+P) represents the fraction of detections, which are events:

(4) $$+P=\frac{TP}{TP+FP}\times 100\%$$

And the detection error rate (DER):

(5) $$\mathrm{Der}=\frac{FP+FN}{TP+FN}\times 100\%$$

where TP is the number of True Positive beats (correctly detected), FN is the number of False Negative beats (erroneously missed), and FP is the number of False Positive beats (erroneously detected) [23].

For comparison, the heart beats information in the annotation file of each recording is read for reference using the WFDB tool [24]. During a beat-by-beat comparison, reference beat labels in the annotation file and detected R points are matched by pairs. A match case is counted where the absolute value of the time difference between the detected R point and the reference is less than 150 ms [23].

Table 1 is the resulting performance of the proposed algorithm tested by the MLII lead and V5 lead of all 48 recordings in the MIT-BIH arrhythmia database. Segments of data in which ventricular flutter or fibrillation (VF) is present are only excluded from beat-by-beat comparisons (for QRS and VEB detection) [23]. A total of 109,443 beats were tested in total and Se is 99.82%, + P is 99.81%, and Der is 0.36% when applying the proposed method. Table 2 reports the performance of the proposed method in different rhythms using the annotation files of the MIT-BIH arrhythmia database. The QRS detection sensitivity of the PVC rhythms is 98.95%, which is sufficient for mobile health applications.

Table 3 reports the function and performance comparison of the proposed method with other methods reported from the original published papers using the MIT-BIH arrhythmia database. As shown in Table 3, five products lead to embedded applications. Among them, the proposed approach is the only product which recognizes four types of point R and is able to provide a preliminary heart rhythm classification in embedded devices. The comparison implies that the proposed real-time method has a high sensitivity (Se) and positive prediction (+P), but low detection error rate (DER). Furthermore, the proposed method not only detects the QRS waves, but also detects the R points in various waveforms with a very low computational effort, which is suitable for wearable device applications and meets the requirement for new generation ECG products.

## 4. Implementation

### 4.1. Implementation for a Mobile Phone

**Figure 14:**
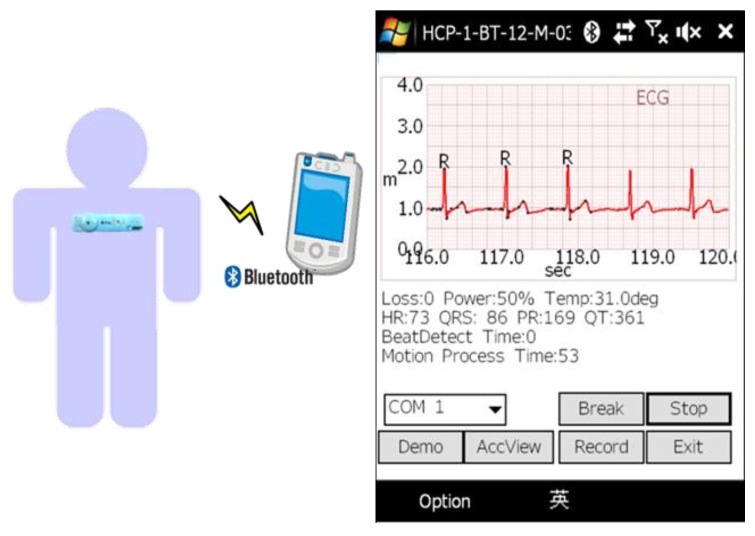
The graphic user interface and algorithm implementation for a mobile healthcare system.

**Figure 15:**
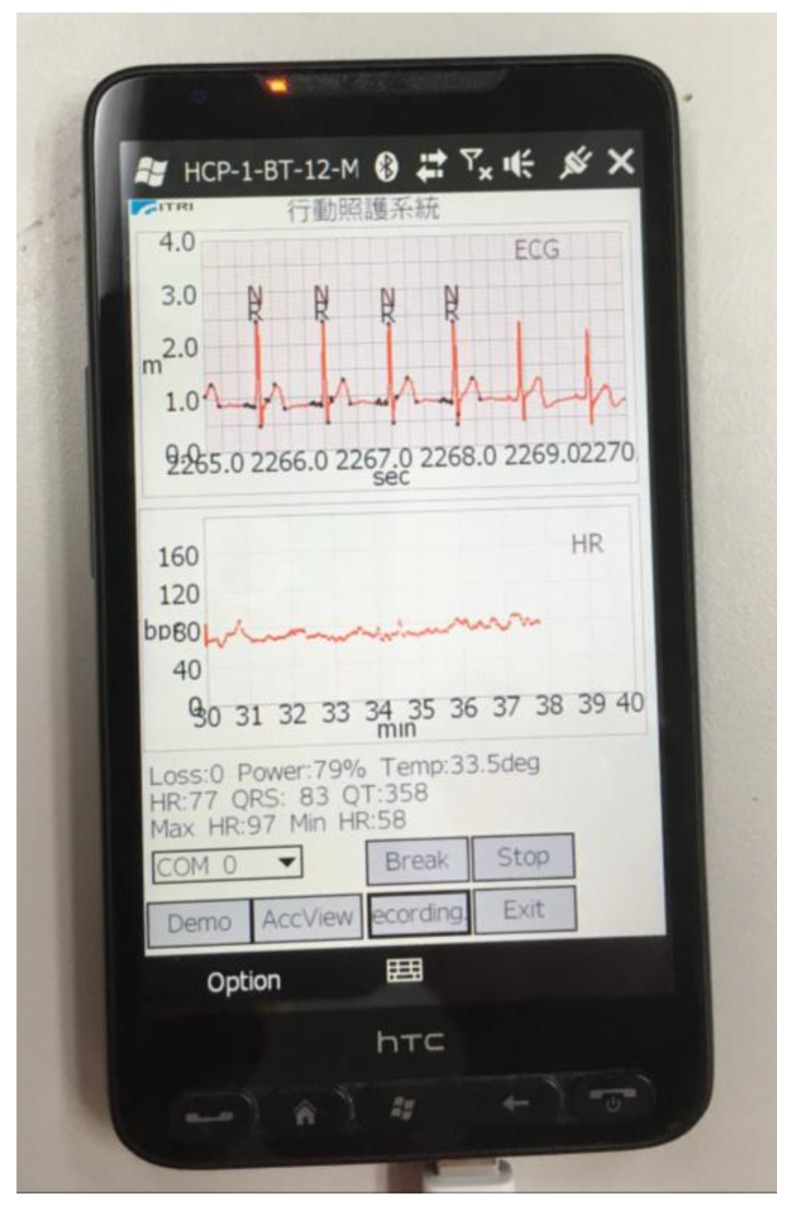
Implementation of daily ECG and heartrate monitoring using the proposed algorithm.

The algorithm has been implemented in a windows mobile phone for a mobile healthcare system and is shown in Figure 14. The mobile phone has a 528 MHz CPU and a Bluetooth module to receive the ECG signal from a wearable ECG patch with a 120 Hz sampling rate, where the ECG patch is developed by the Industrial Technology Research Institute (ITRI). In the system, the graphic user interface (GUI) and proposed algorithm are coded by Microsoft Visual C++. The average time cost of running the proposed algorithm in the mobile phone is less than 1 milliseconds. To verify the stability of the proposed algorithm, the implementation of daily ECG and heartrate monitoring are carried out as shown in Figure 15.

### 4.2. Implementation in an Embedded System

**Figure 16:**
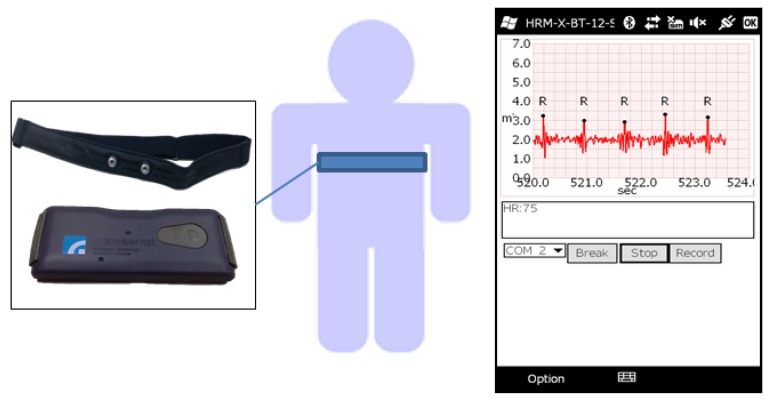
The algorithm implementation in a wearable heart rate sensor.

To implement the healthcare IoT system, an efficient and low computational complexity algorithm is necessary to reduce transition envelopes and save storage space. The proposed algorithm is implemented in a wearable heart rate sensor developed by ITRI, shown in Figure 16, which contains a Corex-M3 MCU running at a frequency of 24 MHz. The average time cost of running the proposed algorithm in the Cortex-M3 MCU is less than 10 milliseconds.

**Figure 17:**
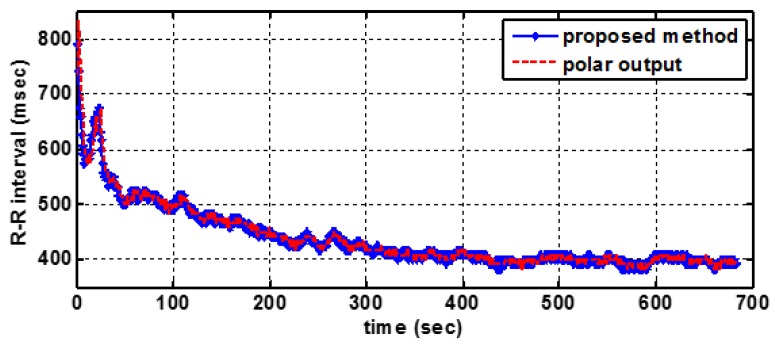
A sample R-R Interval curves.

To demonstrate the resulting performance in a running activity, a market product of the Polar heart rate sensor [28] is used as an R-R interval (RRI) reference. There are 10 RRI data of 10 different persons running on the treadmill, which are collected by the proposed system and the Polar product at the same time. Figure 17 illustrates a good consistency between the results obtained by both systems. Table 4 shows that the max RRI difference is 2.3 ms and the average RRI difference of the 10 people is less than 1 ms, which demonstrates the flexibility of the proposed method to heart rate real-time detection.

### 4.3. A Study Case of Walking

The motion artifact issue has been taken into consideration in this study. The testing database, MIT-BIH arrhythmia database, was recorded from an ambulatory ECG device and the testing results using the proposed algorithm have been listed in Table 1 and Table 3. Furthermore, in our clinical study data using the wearable ECG device mentioned in the article, a study case of ‘walking’ is suitable to demonstrate the noise immunity of the proposed method.

**Figure 18:**
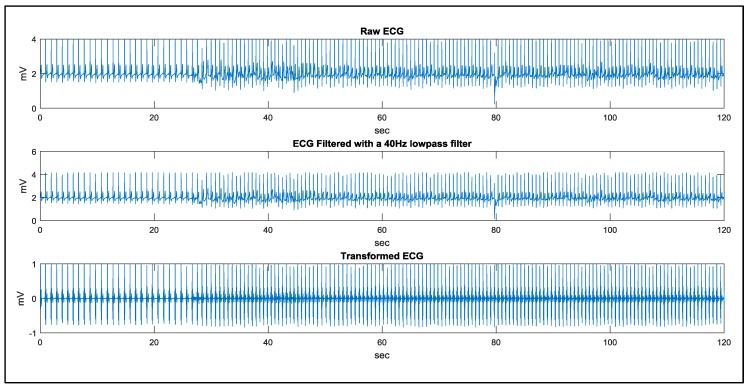
The algorithm implementation in a wearable heart rate sensor.

**Figure 19:**
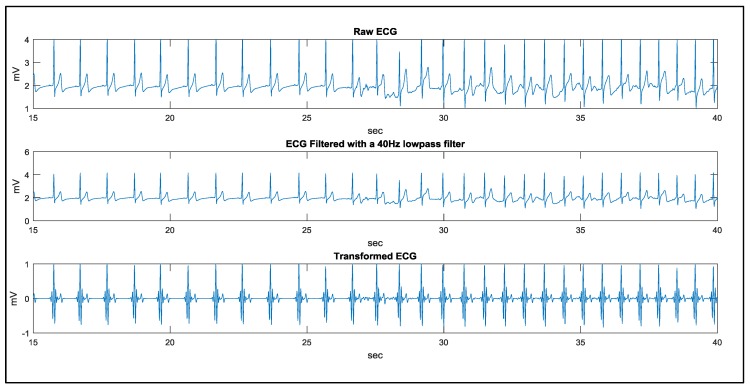
The signal processing results from 15 to 40 s.

**Figure 20:**
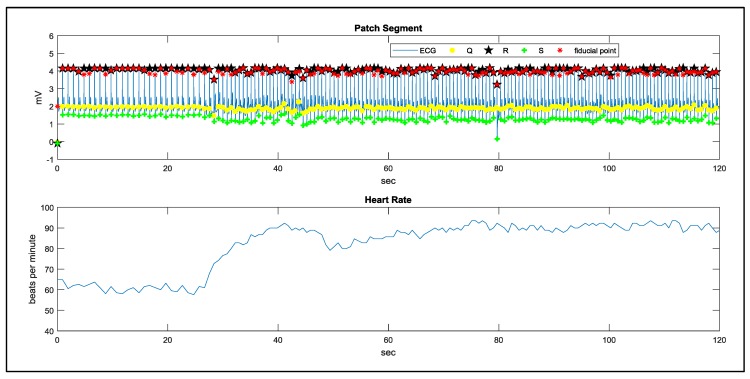
The results of QRS detection, R point recognition, and heart rate curve.

In the study case of walking, a man remains static in the first 20 s and starts walking in the following 100 s. The signal processing results using the proposed method are demonstrated in Figure 18 and Figure 19. The results of QRS detection, R point recognition, and the calculated heart rate curve are demonstrated in Figure 20. In order to evaluate the noise immunity of the proposed method, we have used the SNR improvement measure given by:

(6) $$SNR_{imp}[\mathrm{dB}]=SNR_{output}-SNR_{input}=10\log\left[\frac{\sum_{i}{\left|X_{n}\left(i\right)-X\left(i\right)\right|}^{2}}{\sum_{i}{\left|X_{d}\left(i\right)-X\left(i\right)\right|}^{2}}\right]$$

where X denotes the clean ECG, $X_{d}$ is the denoised signal, and $X_{n}$ represents the noisy ECG [29]. In this study, we assign:

X: the filtered signal in static
$X_{d}$: the filtered signal in walking
$X_{n}$: the raw signal in walking

The average SNR improvement is around 3.53 dB. In practice, in order to avoid too much computing effort during the de-noise process, we also use a well-designed fixing mechanism to reduce the motion artifact, and the proposed algorithm is thus sufficient to process signals satisfactory for the 120 test clinical study.

## 5. Conclusions

A real-time QRS detection and R point recognition method for a wearable single lead ECG device was proposed, which consisted of a signal transform, QRS fiducial point detection, and R point recognition. The proposed method is robust and insensitive to baseline wandering, amplitude variation, and atypical QRS waveforms. Verification using the MIT-BIH arrhythmia benchmark demonstrates that the detected sensitivity (Se), positive prediction (+P), and detection error rate (DER) of the proposed method are as good as those reported during existing approaches. The motion artifact issue has been taken into consideration in this study. The testing database, MIT-BIH arrhythmia database, was recorded from an ambulatory ECG device and testing results using the proposed algorithm have been listed in Table 1 and Table 3. A study case of ‘walking’ is also demonstrated. In practice, in order to avoid too much computing effort during the de-noise process, we also use a well-designed fixing mechanism to reduce the motion artifact, and the proposed algorithm is thus sufficient to process signals satisfactory for the 120 test clinical study. In conclusion, the proposed method meets the requirement of real-time applications on a mobile phone or an embedded system for new-generation ECG devices, owing to its computational simplicity and efficiency. The real time performance of the proposed work is shown in Table 5, where implanted platforms with corresponding processing times are provided in detail. This work is the only one among the references in Table 3 that provides quantitative data as a benchmark for further studies.

## Figures and Tables

**Table 1:** Performance of the proposed method using the single channel of the MIT-BIH arrhythmia database.

No.	Lead	Se%	+P%	DER%	TB	FN	FP
100	MLII	100.00	100.00	0.00	2271	0	0
101	MLII	99.89	99.79	0.32	1864	2	4
102	V5	100.00	100.00	0.00	2186	0	0
103	MLII	100.00	100.00	0.00	2083	0	0
104	V5	99.64	99.11	1.25	2228	8	20
105	MLII	99.53	98.84	1.61	2571	12	30
106	MLII	100.00	99.95	0.05	2026	0	1
107	MLII	99.81	100.00	0.19	2136	4	0
108	MLII	99.38	98.21	2.40	1762	11	32
109	MLII	99.92	100.00	0.08	2531	2	0
111	MLII	99.95	99.91	0.14	2123	1	2
112	MLII	100.00	99.96	0.04	2538	0	1
113	MLII	100.00	100.00	0.00	1793	0	0
114	MLII	100.00	100.00	0.00	1878	0	0
115	MLII	100.00	100.00	0.00	1952	0	0
116	MLII	99.38	99.75	0.87	2411	15	6
117	MLII	100.00	100.00	0.00	1534	0	0
118	MLII	100.00	99.91	0.09	2277	0	2
119	MLII	100.00	100.00	0.00	1986	0	0
121	MLII	99.89	100.00	0.11	1862	2	0
122	MLII	100.00	100.00	0.00	2475	0	0
123	MLII	100.00	100.00	0.00	1517	0	0
124	MLII	100.00	100.00	0.00	1618	0	0
200	MLII	99.92	99.35	0.73	2600	2	17
201	MLII	99.29	100.00	0.71	1962	14	0
202	MLII	99.86	99.95	0.19	2135	3	1
203	MLII	98.79	99.06	2.13	2979	36	28
205	MLII	99.74	99.96	0.30	2655	7	1
207	MLII	99.62	99.36	1.02	1859	7	12
208	MLII	99.46	99.76	0.78	2954	16	7
209	MLII	99.97	99.93	0.10	3004	1	2
210	MLII	98.94	99.81	1.24	2649	28	5
212	MLII	100.00	100.00	0.00	2747	0	0
213	MLII	99.91	100.00	0.09	3249	3	0
214	MLII	99.96	99.96	0.09	2261	1	1
215	MLII	100.00	100.00	0.00	3362	0	0
217	MLII	99.91	99.95	0.14	2207	2	1
219	MLII	100.00	99.91	0.09	2153	0	2
220	MLII	100.00	100.00	0.00	2047	0	0
221	MLII	99.84	100.00	0.16	2426	4	0
222	MLII	99.76	100.00	0.24	2482	6	0
223	MLII	99.96	100.00	0.04	2604	1	0
228	MLII	99.81	98.99	1.21	2052	4	21
230	MLII	100.00	99.91	0.09	2255	0	2
231	MLII	100.00	100.00	0.00	1570	0	0
232	MLII	100.00	99.72	0.28	1779	0	5
233	MLII	99.97	100.00	0.03	3078	1	0
234	MLII	100.00	100.00	0.00	2752	0	0
Total		99.82	99.81	0.36	109,443	193	203

**Table 2:** Performance during different rhythms using the MIT-BIH arrhythmia database.

Rhythm Type	Symbol	TB	TP	FN	Se%
Normal beat	N	75,013	74,939	74	99.90
Left bundle branch block beat	L	8072	8067	5	99.94
Right bundle branch block beat	R	7256	7256	0	100.00
Atrial premature beat	A	2544	2543	1	99.96
Aberrated atrial premature beat	A	150	133	17	88.67
Nodal (junctional) premature beat	J	83	83	0	100.00
Supraventricular premature beat	S	2	2	0	100.00
Premature ventricular contraction	V	7130	7055	75	98.95
Fusion of ventricular and normal beat	F	803	795	8	99.00
Atrial escape beat	E	16	16	0	100.00
Nodal (junctional) escape beat	J	229	229	0	100.00
Ventricular escape beat	E	106	106	0	100.00
Paced beat	/	7024	7017	7	99.90
Fusion of paced and normal beat	F	982	981	1	99.90
Unclassifiable beat	Q	33	28	5	84.85
Total		109,443	109,250	193	99.82

**Table 3:** Comparison of this study with other studies using the MIT-BIH arrhythmia database.

Method	Types of Point R Recognized	Embed. App.	Se%	+P%	DER%	TB	TP	FN	FP
The proposed approach	4	Y	99.82	99.81	0.36	109,443	109,250	193	203
Pan and Tompkins [5]	*	Y	99.76	99.56	0.68	116,137	115,860	277	507
Hamilton and Tompkins [6]	*	Y	99.69	99.77	0.54	109,267	108,927	340	248
Farashi [7]	2	N	99.75	99.85	0.40	109,965	109,692	273	163
Phukpattaranont [8]	2	N	99.82	99.81	0.38	109,483	109,281	202	210
Manikandan and Soman [9]	1	N	99.93	99.87	0.20	109,496	109,417	79	140
Merah et al. [12]	1	N	99.84	99.88	0.28	109,494	109,316	178	126
Cristov [14] (algorithm 2)	*	N	99.78	99.78	0.44	110,050	109,615	240	239
Karimipour and Homaeinezhad [15]	*	N	99.81	99.70	0.43	116,137	115,945	192	308
Dohare et al. [17]	1	N	99.21	99.34	1.45	109,966	109,096	870	728
Sharma and Sunkaria [18]	2	N	99.50	99.56	0.93	109,488	108,979	509	428
Yazdani and Vesin [21]	2	Y	99.87	99.90	0.22	109,494	109,357	137	108
Castells-Rufas and Carrabina [25]	*	Y	99.43	99.67	0.88	109,494	108,880	614	353
Jinkwon Kim and Hangsik Shin [26]	*	N	99.90	99.91	0.19	109,494	109,357	107	97
DS Benitez et al. [27]	1	N	99.81	99.83	0.36	N/A	N/A	203	187

* Only QRS complexes detection.

**Table 4:** The average R-R interval of 10 test cases.

ID	(This Study) Average RRI (ms)	(Polar Output) Average RRI (ms)	Difference (ms)
P201009271648	488.2	489	0.8
P201011171707	499.6	499.6	0
P201011161440	514.4	515	0.6
P201011171720	457.3	457.8	0.5
P201011171149	425.7	428	2.3
P201011171731	528.7	529.4	0.7
P201011171742	419.8	420.4	0.6
P201011171805	437.9	438.9	0.9
P201011231627	424.1	424.6	0.5
P201011241604	423.1	423.6	0.5
Average Difference			0.74

**Table 5:** Implanted platforms with the corresponding processing times demonstrated in this study.

Functions	Data	Platform	Processing Time	Coding Tool
	MIT-BIH arrhythmia database (360 Hz)	windows 7 PC with Intel i5 dual core CPU @ 2.5 GHz	Max < 15 ms Average < 6 ms	Matlab (not be optimized)
QRS complex detection, features detection (Q, R, S), 4 types of point R recognition	Real captured ECG signal (120 Hz)	windows mobile phone with 528 MHz CPU	Average < 1 ms	Visual Studio C++
	Real captured ECG signal (120 Hz)	ARM Cortex-M3 MCU running in the frequency of 24 MHz	Average < 10 ms	Keil C
